# A study of factors influencing access to MDT among patients with stage III to stage IV colorectal cancer: a national multicenter survey

**DOI:** 10.3389/fonc.2026.1646284

**Published:** 2026-05-14

**Authors:** Xiao-Hui Wang, Zu-Zhi Wang, Hui-Fang Xu, Hong-Wei Liu, Dong-Ling Wang, Chang-Yan Feng, Xi Zhang, Wen-Jun Wang, Yan-Qin Yu, Shuang-Xia Duan, Ji-Hai Shi, Yu-Qian Zhao, Ma Li, Yun-Yong Liu, Juan-Xiu Huang, Yan-Ping Fan, Ji Cao, Xiao-Fen Gu, Li Li, Xue-Mei Lian, Jing-Chang Du, You-Lin Qiao

**Affiliations:** 1 Gansu Provincial Cancer Hospital, Lanzhou, China; 2 School of Public Health, Gansu University of Chinese Medicine, Lanzhou, China; 3 The Affiliated Cancer Hospital of Zhengzhou University & Henan Cancer Hospital, Henan Engineering Research Center of Cancer Prevention and Control, Henan International Joint Laboratory of Cancer Prevention, Zhengzhou, China; 4 Chongqing Key Laboratory of Translational Research for Cancer Metastasis and Individualized Treatment, Chongqing University Cancer Hospital, Chongqing, China; 5 Key Laboratory of Carcinogenesis and Translational Research (Ministry of Education/Beijing), Beijing Office for Cancer Prevention and Control, Peking University Cancer Hospital & Institute, Beijing, China; 6 School of Nursing, Jining Medical University, Jining, China; 7 The Department of Public Health and Preventive Medicine, The Research Center of Clinical epidemiology, Baotou Medical College, Baotou, Inner Mongolia, China; 8 Xinxiang Central Hospital, Xinxiang, China; 9 Sichuan Cancer Hospital & Institute, Sichuan Cancer Center, School of Medicine, University of Electronic Science and Technology of China, Chengdu, China; 10 Public Health School, Dalian Medical University, Dalian, China; 11 Liaoning Office for Cancer Control and Research, Cancer Hospital of China Medical University, Liaoning Cancer Hospital and Institute, Shenyang, China; 12 Wuzhou Red Cross Hospital, Wuzhou, China; 13 State Key Laboratory of Oncology in South China, Collaborative Innovation Center for Cancer Medicine, Sun Yat-sen University Cancer Center, Guangzhou, China; 14 The First Affiliated Hospital of Guangxi Medical University, Nanning, Guangxi, China; 15 Affiliated Tumor Hospital, Xinjiang Medical University, Urumqi, Xinjiang, China; 16 Department of Clinical Research, The First Affiliated Hospital, Jinan University, Guangzhou, Guangdong, China; 17 School of Public Health and Management, Chongqing Medical University, Chongqing, China; 18 College of Public Health, Chengdu Medical College, Chengdu, China

**Keywords:** factors, MDT, quality of life, stage III to IV CRC, treatment modalities

## Abstract

**Background and objective:**

The disease burden of colorectal cancer (CRC) in China is relatively heavy, and although the multidisciplinary treatment (MDT) model can improve efficacy and resource efficiency, there are inequalities in its accessibility. The aim of this study was to investigate the key factors affecting access to MDT among patients with stage III to stage IV CRC.

**Methods:**

A multi-stage stratified sampling method is adopted to conduct a cross-sectional survey of 4,589 stage III-IV colorectal cancer (CRC) patients across 19 hospitals nationwide.

**Results:**

Only 796 (17.3%) of the 4,589 patients with stage III-IV CRC included in this study participated in MDT, with more males (61.4%) than females (38.6%). Univariate analysis showed that:patients’ location, medical visits to provincial-capital-city-hospitals and prefecture-level-city-hospitals, education background, number of medical institutions been visited, family income, patients’ circumstance of disease, initial diagnosis stage, whether metastasis occurred at initial diagnosis, endoscopic interventional therapy, chemotherapy, targeted therapy, traditional Chinese medicine(TCM) or palliative treatment, and treatment efficacy (p<0.05) are factors influencing patient participation in MDT. Multivariate analysis shows that patients in North China (OR: 2.69, 95% CI: 1.98-3.67), Central China (OR: 1.59, 95% CI: 1.15-2.21), Southwest China (OR: 5.19, 95% CI: 3.78-7.11), and Northwest China (OR: 2.28, 95% CI: 1.51-3.43) are more likely to participate in MDT. Patients treated at city-level hospitals (OR: 1.84, 95% CI: 1.50-2.26) and those receiving care at two hospitals (OR: 1.28, 95% CI: 1.02-1.61) also show higher MDT participation rates. Those in the recurrent treatment phase (OR: 3.21, 95% CI: 1.56-6.58) are significantly more likely to engage in MDT. Additionally, patients undergoing chemotherapy (OR: 2.62, 95% CI: 1.75-3.93), targeted therapy (OR: 1.38, 95% CI: 1.10-1.73), or traditional Chinese medicine (TCM)/palliative treatment (OR: 1.76, 95% CI: 1.41-2.22) have an increased likelihood of MDT participation. There is a positive association between participation in MDT and the overall health-related quality of life (HRQOL) score in colorectal cancer patients.

**Conclusion:**

Different regions, healthcare organizations, patients’ stage of treatment, modalities of treatment and quality of life influence access to MDT for patients with Stage IV CRC, while MDT care is significantly associated with better quality of life.

## 1 Introduction

Colorectal cancer (CRC) is one of the most prevalent malignant tumors worldwide, with high incidence and mortality rates (1). This trend is particularly pronounced in emerging economies and younger populations (2, 3). According to the latest statistics published by the World Health Organization in 2022 (1), there were 1.926 million new CRC cases globally, accounting for 9.6% of all malignant tumor incidences and ranking third in all cancer incidence rates, with 904,000 deaths, representing 9.3% of all cancer-related deaths and ranking second in all cancer mortality rates.

In China, CRC imposes a significant disease burden. The 2022 data (4) indicates that 517,000 new CRC cases were reported, making up 26.9% of all incident cancer cases. CRC-related deaths reached 240,000, accounting for 9.3% of total cancer mortality and ranking fourth in all cancer mortality rates. Moreover, most CRC cases in China are diagnosed at stage IV, where disease severity leads to poorer quality of life for patients (5).

The multidisciplinary treatment (MDT) model focuses on patient-centered, individualized, and optimized treatment strategies (6). MDT has been shown to improve survival rates for CRC surgery patients, enhance quality of life, reduce complications, and increase healthcare resource efficiency (7–9). China has been actively promoting MDT development; however, accessibility varies across different regions and demographics. There is currently limited research on the key factors influencing patients’ access to MDT services. This study aims to analyze the determinants affecting MDT accessibility for stage III to stage IV CRC patients, to promote the popularization and optimization of the MDT model, to benefit more CRC cancer patients, and to provide a basis for the development of health policies.

## 2 Materials and methods

### 2.1 Study design

This study used a national multi-center cross-sectional survey design, which has been discussed in previous literature (10).The aim is to systematically analyze the factors influencing access to MDT treatment in Chinese patients with stage III to IV CRC. Based on China’s traditional administrative division system, the study divided mainland China into seven geographic regions (North China, Northeast China, Northwest China, Central China, East China, South China, and Southwest China) (11). Multi-stage stratified sampling was adopted to determine the participating hospitals. In stage I, 2 cities of each region were selected by simple random sampling. In stage II, a tertiary cancer hospital and/or a general hospital was selected from each city. A total of 19 hospitals (10 tertiary cancer hospitals and 9 general hospitals) were selected.The study was approved by the Institutional Review Board of Cancer Hospital of Henan Province (Approval No. 2019273), and other hospitals were informed and consented to the study. Written informed consent was obtained from all participating patients before the study was conducted, and the ethical guidelines of the Declaration of Helsinki were strictly followed (as revised in 2013).

### 2.2 Subjects of the study

According to the 8th edition of the American Joint Committee on Cancer (AJCC) tumor-node-metastasis (TNM) staging system (12). We selected patients with colorectal cancer who had received a definitive histopathological diagnosis from the participating hospitals. Based on this diagnosis, we performed clinical TNM staging assessments using imaging studies and physical examinations, ultimately identifying patients with stage III or IV colorectal cancer. The CRC patients were coming from the inpatients, who were recruited by the trained interviewers. The inclusion criteria were as follows: confirmed stage III or IV CRC patients; aged ≥18 years; without any dementia, language communication disorder and able to understand the investigation procedure. The exclusion criteria were as follows: severe physical, cognitive, and/or verbal impairments that would interfere with a patient’s ability to complete the questionnaire.The respondents were recruited from the Department of Medical Oncology, Surgical Oncology, Department of Radiotherapy and Department of Anorectal Surgery of the selected hospitals. All eligible patients signed informed consent before enrollment.

### 2.3 Basis of sample size calculation and actual recruitment

There are approximately 400,000 patients with advanced colorectal cancer (CRC) in China. Taking into account the feasibility of conducting a nationwide multicenter cross-sectional survey and the need for regional representativeness, and factoring in an estimated non-response rate of approximately 10%, this study set a minimum effective sample size of 4,445 cases. Ultimately, 4,589 cases were included in the final analysis.

### 2.4 Data collection

This study employed standardized self-report questionnaires and semi-structured interviews (SSQ) that had been validated for reliability and validity (10, 13). The questionnaire covered various aspects, including the medical history of late-stage colorectal cancer patients, current treatment status, quality of life, medical expenses and reimbursements, as well as potential factors influencing access to MDT.All survey investigators underwent standardized training and certification, which included guidance on questionnaire interpretation, communication skills, and medical ethics compliance. Raw data were entered by two trained research assistants using EpiData software version 3.1 (EpiData Corporation, Redwood City, CA, USA). All data cleaning and analysis was done in the Statistical Analysis System version 9.3 (SAS Institute, Cary, NC, USA). Surveys with a completion rate of over 95% are considered valid. Researchers will conduct quality control on 3% of the questionnaires from 14 centers nationwide using a telephone survey method. At the same time, the heads of each center will conduct quality control on a 5% sample of the questionnaires collected by their respective investigators from colorectal cancer patients and healthcare professionals. Based on the widely validated Chinese Functional Assessment of Cancer Therapy-Colorectal (FACT-C) and the European Organization for Research and Treatment of Cancer (EORTC QLQ-C30) questionnaire, this study developed a new questionnaire called FACT-C plus QLQ-C9 for this survey to measure patients’ HRQOL. It consists primarily of six functional subscales (physical, social/family, emotional, performance, colorectal cancer, and cognitive), two symptom subscales (fatigue and sleep disturbances), and an item regarding financial difficulties.In this study, patients may participate in discussions and decision-making regarding personalized treatment plans under the guidance of their physicians. MDT care is primarily conducted through regular multidisciplinary tumor board meetings and joint multidisciplinary consultations, with treatment strategies developed collaboratively by relevant disciplines. Further details regarding the SSQ and FACT-C plus QLQ-C9 can be found in previously published studies (10).

### 2.5 Statistical analyses

Qualitative data were demonstrated as frequencies (n) and constitutive ratios (%), quantitative data were parametrically described using mean ± standard deviation ( $\bar{\text{x}}$ ± s) after the Shapiro-Wilk normality test (W = 0.96, P = 0.082), and median (M) and interquartile spacing (IQR) were reported for skewed distribution data. One-way analyses of categorical variables were performed using chi-square tests, and continuous variables were analyzed using ANOVA or t-tests. Covariates with P<0.05 in univariate analysis were included in multivariate logistic regression models for further analysis. All statistical analyses were analyzed using IBM SPSS software (Version 26.0) with a significance level of 0.05.

## 3 Results

### 3.1 Basic characteristics of patients with stage III to IV CRC participating in the MDT

Among the 4,589 patients with stage III to IV CRC included, 796 (17.3%) participated in MDT team management. There were more males (61.4%) than females (38.6%), retirees (27.8%) and agriculture, forestry, animal husbandry and fishery workers (23.1%) were the main occupational groups, more than 50% had junior high school education or above, the health insurance coverage rate was 98.7%, and 99.6% of the patients chose to seek medical treatment in a different location, and the proportion of patients with colorectal/rectal cancers was 47.4% and 51.6%, respectively. (**Table 1**).

**Table 1:** Basic characteristics of patients with stage III to IV colorectal cancer participating in MDTs.

Diagnostic characteristics	Number of patients	Proportion (%)
Identity of respondents		
Patient	596	74.9
Relatives	200	25.1
Gender		
Male	486	61.4
Female	307	38.6
Careers		
Public servants and enterprise employees	46	5.8
Professional and technical staff	36	4.5
Clerical and related personnel	26	3.3
Commercial and service workers	15	1.9
Laborers in agriculture, forestry, animal husbandry and fishery industries	184	23.1
Workers in transport, construction, production, manufacturing, etc.	46	5.8
Military personnel	2	0.3
Freelancers/self-employed	83	10.4
Student	1	0.1
Housewife	72	9.0
Retirement (from work)	221	27.8
Laid-off, unemployed	18	2.3
Others	46	5.8
Marital status		
Married	746	93.7
Unmarried	11	1.4
Divorced from (one’s spouse)	12	1.5
Bereaved of one’s spouse (literary)	27	3.4
Type of disease		
Colon	377	47.4
Rectum	411	51.6
Other, please specify	8	1.0
Level of education*		
No formal education	65	8.2
Elementary schools	178	22.4
Junior high school	244	30.7
Senior high school	135	17.0
Middle level vocational school	29	3.6
High level vocational school	69	8.7
University undergraduate	69	8.7
Master’s degree or above	6	0.8
Genetic testing		
Yes	507	63.7
No	230	28.9
Currently unknown	59	7.4
health insurance*		
Yes	784	98.7
No	10	1.3
Medical treatment in a different place		
Yes	793	99.6
No	3	0.4
Hospital level		
Hospitals in first-tier cities above the provincial level	316	39.7
Provincial capital city hospitals	256	32.2
Prefectural city hospitals	534	67.1
County hospitals	220	27.6

*Indicates partial missing data values.

### 3.2 Analysis of factors influencing patient participation in MDTs

#### 3.2.1 Univariate logistic regression analysis of patient participation in MDT

According to the results of the univariate logistic regression analysis, factors influencing patient participation in Multidisciplinary Treatment (MDT) management include geographical region, treatment at provincial or city-level hospitals, education level, number of healthcare institutions visited, household income, disease stage, cancer staging at initial diagnosis, presence of metastasis at first diagnosis, endoscopic interventional therapy, chemotherapy, targeted therapy, traditional Chinese medicine or palliative treatment, and treatment efficacy. All factors showed statistical significance (p<0.05) (**Table 2**).

**Table 2:** Univariate analysis of participation in MDT in patients with stage III to IV CRC.

Variables	Total cases n (%)	Number of multidisciplinary consultations		χ^2^	P
		Yes (%)	No (%)		
	4589	796	3793		
Region				247.995	<0.001
Eastern China	1319 (28.7)	160 (20.1)	1159 (30.6)		
Northern China	565 (12.3)	140 (17.6)	425 (11.2)		
South China	672 (14.6)	76 (9.5)	596 (15.7)		
Central China	690 (15.0)	91 (11.4)	599 (15.8)		
North-eastern	364 (7.9)	32 (4.0)	332 (8.8)		
Southwestern	652 (14.2)	234 (29.4)	418 (11.0)		
Northwestern	327 (7.1)	63 (7.9)	264 (7.0)		
Hospital level					
A:Attendance at a hospital in a first-tier city at or above the provincial level				0.768	0.087
Yes	1846 (40.2)	316 (39.7)	1530 (40.3)		
No	2743 (59.8)	480 (60.3)	2263 (59.7)		
B:Attendance at hospitals in provincial capitals				5.955	0.015
Yes	1310 (28.5)	256 (32.2)	1054 (27.8)		
No	3279 (71.5)	540 (67.8)	2739 (72.2)		
C:Attendance at prefecture-level city hospitals				30.05	<0.001
Yes	2676 (58.3)	534 (67.1)	2142 (56.5)		
No	1913 (41.7)	262 (32.9)	1651 (43.5)		
D:Attendance at a county hospital				0.272	0.602
Yes	1306 (28.5)	220 (27.6)	1086 (28.6)		
No	3283 (71.5)	576 (72.4)	2707 (71.4)		
Patient’s age				3.204	0.201
<45	464 (10.1)	83 (10.4)	381 (10.0)		
40~60	1775 (38.7)	328 (41.2)	1447 (38.1)		
≥60	2350 (51.2)	385 (48.4)	1965 (51.8)		
Gender				1.411	0.235
Male	2730 (59.5)	489 (61.4)	2241 (59.1)		
Female	1859 (40.5)	307 (38.6)	1552 (40.9)		
Marital status*				0.539	0.97
Married	4318 (94.1)	746 (93.7)	3572 (94.2)		
Unmarried	62 (1.4)	11 (1.4)	51 (1.3)		
Divorced from (one’s spouse)	66 (1.4)	12 (1.5)	54 (1.4)		
Bereaved of one’s spouse (literary)	142 (3.1)	27 (3.4)	115 (3.0)		
Highest level of Education*				20.601	0.008
No formal education	388 (8.5)	65 (8.2)	323 (8.5)		
Elementary schools	942 (20.5)	178 (22.4)	764 (20.1)		
Junior high school	1478 (32.2)	244 (30.7)	1234 (32.5)		
Senior high school	762 (16.6)	135 (17.0)	627 (16.5)		
Middle level vocational school	282 (6.1)	29 (3.6)	253 (6.7)		
High level vocational school	408 (8.9)	69 (8.7)	339 (8.9)		
University undergraduate	294 (6.4)	69 (8.7)	225 (5.9)		
Master’s degree or above	32 (0.7)	6 (0.8)	26 (0.7)		
Participation in health insurance*				0.338	0.844
Yes	4525 (98.6)	784 (98.5)	3741 (98.6)		
No	56 (1.2)	10 (1.3)	46 (1.2)		
Type of disease*					
Colon	2063 (45.0)	377 (47.4)	1686 (44.5)	2.465	0.456
Rectum	2470 (53.8)	411 (51.6)	2059 (54.3)		
The rest	56 (1.2)	8 (1.0)	48 (1.2)		
Number of hospitals visited*				34.646	<0.01
1	1334 (29.1)	176 (22.1)	1158 (30.5)		
2	2327 (50.7)	436 (54.8)	1891 (49.9)		
≥3	840 (18.3)	178 (22.4)	662 (17.5)		
Household income*				14.7	0.012
Basically no income	763 (16.6)	152 (19.1)	611 (16.1)		
<50,000	1861 (40.6)	293 (36.8)	1568 (41.3)		
50,000~ 100,000	1293 (28.2)	226 (28.4)	1067 (28.1)		
100,000~200,000	523 (11.4)	88 (11.1)	435 (11.5)		
>200,000	133 (2.9)	35 (4.4)	98 (2.6)		
Availability medical care in different city				1.081	0.299
Yes	755 (99.8)	793 (99.6)	3788 (99.9)		
No	8 (0.2)	3 (0.4)	5 (0.1)		
Disease stage of the patient*				246.49	<0.01
Diagnosed but not yet started any treatment	157 (3.4)	12 (1.5)	145 (3.8)		
Failure to change programme at first treatment	2088 (45.5)	239 (30.0)	1849 (48.7)		
First Treatment Replacement Programme	532 (11.6)	106 (13.3)	426 (11.2)		
Stages of treatment after relapse	1138 (24.8)	362 (45.5)	776 (20.5)		
Recuperation at home with regular reviews (less than 3 years in complete remission)	564 (12.3)	66 (8.3)	498 (13.1)		
Recuperation at home with regular review phase (complete remission for more than 3 years)	106 (2.3)	11 (1.4)	95 (2.5)		
Staging of initial diagnosis*				89.506	<0.01
Phase I	112 (2.4)	8 (1.0)	104 (2.7)		
Phase II	775 (16.9)	83 (10.4)	692 (18.2)		
Phase III	1970 (42.9)	299 (37.6)	1671 (44.1)		
Phase IV	1550 (33.8)	382 (48.0)	1168 (30.8)		
Currently unknown	170 (3.7)	23 (2.9)	147 (3.9)		
First diagnosis of whether metastasis has occurred*				68.056	<0.01
Non-transferable	2854 (62.2)	399 (50.1)	2455 (64.7)		
Metastases to the liver only	641 (14.0)	148 (18.6)	493 (13.0)		
Metastases to the lungs only	179 (3.9)	48 (6.0)	131 (3.5)		
Metastases to both liver and lungs	193 (4.2)	50 (6.3)	143 (3.8)		
Multiple metastases to other sites or throughout the body	696 (15.2)	149 (18.7)	547 (14.4)		
Treatment					
A:Whether or not the patient had surgery during the consultation*				0.443	0.801
Yes	742 (16.2)	133 (16.7)	609 (16.1)		
No	3838 (83.6)	662 (83.2)	3176 (83.7)		
B:Has the patient had an endoscopic intervention during the consultation*				6.776	0.034
Yes	4438 (96.7)	759 (95.4)	3679 (97.0)		
No	142 (3.1)	36 (4.5)	106 (2.8)		
C:Whether the patient had radiotherapy during the consultation*				0.895	0.639
Yes	3575 (77.9)	612 (76.9)	2963 (78.1)		
No	1005 (21.9)	183 (23.0)	822 (21.7)		
D:Whether the patient had chemotherapy during the consultation*				67.218	<0.001
Yes	621 (13.5)	36 (4.5)	585 (15.4)		
No	3959 (86.3)	759 (95.4)	3200 (84.4)		
E:Has the patient had targeted therapy during the consultation*				148.86	<0.001
Yes	3263 (71.1)	425 (53.4)	2838 (74.8)		
No	1317 (28.7)	370 (46.5)	947 (25.0)		
F:Whether the patient has had immunotherapy during the course of the consultation*				2.07	0.355
Yes	4472 (97.5)	771 (96.9)	3701 (97.6)		
No	108 (2.4)	24 (3.0)	84 (2.2)		
G:Whether the patient has received Chinese herbal medicine or palliative care in the course of the consultation*				75.246	<0.001
Yes	3813 (83.1)	579 (72.7)	3234 (85.3)		
No	767 (16.7)	216 (27.1)	551 (14.5)		
First treatment effect*				110.039	<0.001
Good treatment results	2828 (61.6)	2282 (60.2)	546 (68.6)		
Poor treatment results	329 (7.2)	238 (6.3)	91 (11.4)		
Stable condition (no change)	483 (10.5)	387 (10.2)	96 (12.1)		

*Indicates partial missing data values.

#### 3.2.2 Multifactorial regression analysis of patient participation in MDTs

Geographic analyses (using East China as a reference) showed that North China (OR = 2.69, 95%CI: 1.98-3.67), Central China (OR = 1.59, 95%CI: 1.15-2.21), Southwest China (OR = 5.19, 95%CI: 3.78-7.11) and Northwest China (OR = 2.28, 95%CI: 1.51-3.43) All were significant positive influences, indicating that patients in these regions were significantly more likely to participate in MDTs, with the most prominent uplift effect in the Southwest. Regarding hospital level, attending provincial capital city hospitals was a significant negative influencing factor (OR = 0.69, 95%CI: 0.53-0.90), meaning that patients were less likely to participate in MDT than patients in non-provincial capital hospitals, while attending prefecture-level city hospitals was a positive influencing factor (OR = 1.84, 95%CI: 1.50-2.26), and patients were more likely to participate in MDT than patients in non-territorial city hospitals. Among the personal characteristics of patients, secondary education was a negative influencing factor compared with no formal education (OR = 0.49, 95%CI: 0.28-0.86), implying that patients with secondary education were less likely to participate in the MDT, and those who visited two hospitals were more likely to participate in the MDT than those who visited only one hospital (OR = 1.28, 95%CI: 1.02-1.61). 1.61). In terms of treatment stage, patients in the post-relapse treatment stage were significantly more likely to participate in the MDT compared to patients who did not start treatment after diagnosis (OR = 3.21, 95%CI: 1.56-6.58). In addition, patients who received chemotherapy (OR = 2.62, 95%CI: 1.75-3.93), targeted therapy (OR = 1.38, 95%CI: 1.10-1.73), or TCM/palliative care (OR = 1.76, 95%CI: 1.41-2.22) were significantly more likely to participate in MDT (**Table 3**).

**Table 3:** Multifactorial analysis of participation in MDT in patients with stage III to IV CRC.

Variables	β-coefficient	OR (95 CI %)	P-value
Region			
Eastern China	reference group	1	–
Northern China	0.991	2.69 (1.98, 3.67)	<0.001
South China	0.249	1.28 (0.91, 1.80)	0.151
Central China	0.466	1.59 (1.15, 2.21)	0.005
North-eastern	-0.442	0.64 (0.38, 1.10)	0.106
Southwestern	1.646	5.19 (3.78, 7.11)	<0.001
Northwestern	0.822	2.28 (1.51, 3.43)	<0.001
Level of hospitalization			
A:Attendance at hospitals in provincial capitals			
No	reference group	1	–
Yes	-0.377	0.69 (0.53, 0.90)	0.005
B:Attendance at a local or municipal hospital			
No	reference group	1	–
Yes	0.611	1.84 (1.50, 2.26)	<0.001
Educational attainment			
No formal education	reference group	1	–
Elementary schools	-0.049	0.95 (0.66, 1.38)	0.795
Junior high school	-0.132	0.88 (0.61, 1.26)	0.472
General high school	-0.162	0.85 (0.57, 1.26)	0.422
Middle level (e.g. school)	-0.711	0.49 (0.28, 0.86)	0.012
University college	-0.146	0.86 (0.54, 1.38)	0.538
University undergraduate course	0.103	1.11 (0.68, 1.82)	0.682
Master’s degree or above	-0.244	0.78 (0.27,2.25)	0.650
Number of hospitals attended			
1	reference group	1	–
2	0.250	1.28 (1.02,1.61)	0.033
≥3	0.018	1.02 (0.76,1.36)	0.903
Household income			
Basically no income	reference group	1	–
Less than 50,000	-0.212	0.81(0.62,1.05)	0.114
Less than $50,000 - $100,000	0.013	1.01 (0.76,1.36)	0.932
Less than $100,000 - $200,000	-0.254	0.78 (0.53,1.14)	0.199
20 or more	0.462	1.59 (0.91,2.78)	0.106
Disease stage of the patient			
Diagnosed but not yet started any treatment	reference group	1	–
Failure to change programme at first treatment	0.176	1.19 (0.59,2.42)	0.626
First Treatment Replacement Programme	0.729	2.07 (0.99,4.33)	0.052
Stages of treatment after relapse	1.165	3.21 (1.56,6.58)	0.002
Recuperation at home with regular reviews (less than 3 years in complete remission)	0.122	1.13 (0.53,2.39)	0.750
Recuperation at home with regular review phase (complete remission for more than 3 years)	0.044	1.04 (0.39,2.78)	0.930
Staging of the initial diagnosis of the disease			
Phase I	reference group	1	–
Phase II	0.025	1.03 (0.45,2.34)	0.953
Phase III	-0.004	1.00(0.45,2.22)	0.993
Phase IV	0.319	1.38 (0.59,3.20)	0.459
Other than	1.205	3.34 (0.75,14.86)	0.114
Currently unknown	-0.291	0.75 (0.28, 2.03)	0.568
First diagnosis of metastasis			
Non-transferable	reference group	1	–
Metastases to the liver only	0.063	1.07 (0.74, 1.54)	0.734
Metastases to the lungs only	0.145	1.16 (0.70, 1.90)	0.567
Metastases to both liver and lungs	-0.129	0.88 (0.54, 1.43)	0.605
Multiple metastases to other sites or throughout the body	-0.063	0.94 (0.66, 1.33)	0.723
Treatment			
A:Endoscopic interventions			
No	reference group	1	–
Yes	0.475	1.61 (0.99, 2.61)	0.055
B:Chemotherapy			
No	reference group	1	–
Yes	0.965	2.62 (1.75, 3.93)	<0.001
C:Targeted therapies			
No	reference group	1	–
Yes	0.320	1.38 (1.10, 1.73)	0.006
D:Traditional Chinese Medicine (TCM) treatment or palliative care			
No	reference group	1	–
Yes	0.568	1.76 (1.41, 2.22)	<0.001
First treatment effect			
Good treatment results	reference group	1	–
Poor treatment results	-0.011	0.99 (0.74, 1.33)	0.944
No change in treatment effect	-0.068	0.93 (0.71, 1.22)	0.621

### 3.3 Impact of quality of life on participation in MDT before and after treatment

There was a positive association between patients’pre-treatment quality of life in the physical and social dimensions and MDT participation (p < 0.05). (**Table 4**). The difference between the health-related quality of life (HRQOL) of CRC patients who participated in MDT and those who did not was statistically significant, p<0.05 (**Table 5**).

**Table 4:** Impact of pre-treatment quality of life on participation in the MDT.

Quality of life	MDT		t-value	P
	Yes	No		
Number of examples	796	3793		
Physiological status	1.91 (0.44)	1.96 (0.46)	2.76	0.006
Social/family status	4.27 (0.80)	4.21 (0.87)	-2.05	0.040
Emotional status	2.07 (0.78)	2.09 (0.83)	0.66	0.507
Functional status	2.94 (0.74)	2.90 (0.88)	-1.4439	0.149
HRQOL	2.55 (0.33)	2.55 (0.41)	-0.06	0.955

**Table 5:** Effect of participation in MDT on quality of life after treatment.

Quality of life	MDT		t-value	P
	Yes	No		
Number of examples	796	3793		
Physiological status	2.09 (0.48)	2.07 (0.46)	-0.83	0.404
Social/family status	4.33 (0.76)	4.27 (0.82)	-1.87	0.059
Emotional status	2.19 (0.67)	2.17 (0.71)	-0.77	0.441
Functional status	2.94 (0.74)	2.90 (0.88)	-1.44	0.149
HRQOL	2.62 (0.33)	2.59 (0.35)	-2.15	0.032

## 4 Discussion

MDT teams are routinely composed of medical oncologists, surgeons, radiologists, pathologists, and nursing specialists, integrating multidisciplinary expertise (including oncology, radiation therapy, etc.), and their standardized implementation directly affects patients’ survival prognosis and quality of life (9). In this study, we found through a national multicenter survey that not all patients have equal access to MDT services, and it is affected by factors such as geographic distribution, tier of medical institutions, patients’ educational background and treatment characteristics.

In China, colorectal cancer screening coverage remains insufficient, screening methods are not yet standardized, and a comprehensive national screening system has not yet been established (14). Patients often seek medical care only after symptoms appear, by which time the tumor has usually progressed to stage III or IV (5, 15). The incidence rate is significantly higher among men than among women. This disparity is primarily attributed to men’s higher exposure to risk factors such as smoking, alcohol consumption, high-fat diets, and physical inactivity (16). Additionally, lower rates of participation in health checkups and endoscopic screening among men further exacerbate the disease burden. The rapid rise in metabolic risk factors, such as high body mass index, particularly among men, has also intensified this gender disparity and the overall disease burden (17).

Compared with East China, Southwest China had the highest likelihood of patient participation in MDT, followed by North, Northwest, and Central China. This phenomenon may be related to inter-regional medical service models and resource allocation (18). As a key region in the national “Western Development” and “Healthy China” strategies, Southwest China has invested heavily in standardised oncology care in recent years. Regional medical centers represented by the Affiliated Hospital of Southwest Medical University emphasize the standardization of oncology diagnosis and treatment, and achieve the improvement of patient efficacy and efficient use of medical resources through standardized processes, multidisciplinary collaboration, and quality control (19). The remote consultation and two-way referral mechanism based on Internet hospitals in Southwest China optimises resource allocation and promotes the extension of MDT services to the grassroots (20). Research data from the American Society of Clinical Oncology (ASCO) revealed that 56.8% of the 470 county-level hospitals in the four southwestern provinces carry out MDT and 46% set up cancer control centers (21). East China, as a region with concentrated and developed medical resources (22), the traditional diagnosis and treatment model may still occupy a certain position in some hospitals and departments, which may weaken the need for MDT collaboration. East China has a higher commercial insurance coverage rate (23), and patients may be more inclined to independently choose flexible staged treatment rather than the fixed process required by MDT, a hypothesis that needs to be further verified through the study of the correlation between the payment method and the choice of diagnosis and treatment. North China, as a cluster of high-quality medical resources, has more national oncology centers and a wide coverage of tertiary hospitals, and the higher comprehensive diagnostic and treatment capacity of specialists may have weakened the development of MDT diagnosis and treatment; a more likely factor is the strong technological radiation of national medical centers, such as Beijing, which promotes the downward sinking of MDT resources through healthcare consortiums. The large population base and limited quality resources in Central China may have led to the fact that MDT can only be carried out in a small number of tertiary hospitals, and that patients give up participation due to time costs or financial pressures.

The MDT participation rate of capital city hospitals is lower than that of non-capital city hospitals, while prefecture-level city hospitals show a higher tendency to participate. Capital city hospitals gather high-quality medical resources and patients, but there is a tendency of “emphasising hardware rather than collaboration” in the distribution of resources (24). Tertiary hospitals are highly segmented and specialised, for example, surgeons and doctors from other leading departments have higher decision-making power in MDT (25), and although members of other departments participate in the discussion, their opinions may not be fully valued, which leads to a decrease in the efficiency of collaboration. Although the country promotes MDT pilots, but lack of uniform norms, the implementation standard varies from place to place (26), the provincial capital hospitals due to the strong autonomy but rather difficult to balance the flexibility and uniformity, resource enrichment but may inhibit the efficiency of collaboration (27). A study in the UK also found (28) that attendance at MDT meetings declined in mega hospitals due to increased inter-departmental competition. The state actively promotes the expansion and sinking of high-quality medical resources and a balanced regional layout, and prefecture-level city hospitals, as an important part of the construction of regional medical centers, have a relative concentration of medical resources, moderate patient flow, and a relatively light workload for doctors, which enables hospitals to make more efficient use of existing resources and form effective MDT teams when promoting MDT.

The MDT participation rate of patients with secondary education was lower than that of the group without formal education (OR = 0.49), a finding that differs from conventional perceptions. The occupational characteristics of the secondary school group, which is mostly engaged in skilled labour jobs, may lead to passive reliance on medical decision-making. Compared with the uneducated group, who tend to follow the doctor’s advice in the traditional model of medical care (29), middle-aged patients may have a “semi-professional cognitive bias”, which is a combination of basic medical cognition and a lack of systematic medical knowledge, which may lead to conflicting decision-making. This group is often in the transition zone between formal and informal employment, and the time cost of participating in MDT may affect their income stability. Study Shows Reduced Healthcare Participation in Patients with Irregular Occupations (30).

Patients attending two or more hospitals had higher rates of MDT participation than those attending a single institution. Patients requiring multi-institutional consultation may face more complex conditions, and initial assessment at the first hospital may trigger targeted referrals to MDT-qualified hospitals. Studies have shown a high proportion of primary hospital referrals to tertiary hospitals with MDT needs (31). Patients who actively seek secondary care tend to have higher health needs, and their cautious attitude towards treatment options may prompt an MDT request. Health behaviour theory states that repeat visit behaviour is positively associated with patients’ initiative to participate in decision-making (32). Differences in the speciality strengths of different hospitals may force patients to integrate multidisciplinary resources through cross-hospital consultations, which objectively forms a “spontaneous MDT”.

The recurrence stage often faces the problem of synergy between local and systemic therapy. Local recurrence of rectal cancer needs to be weighed against the survival benefit of pelvic contouring versus the patient’s quality of life, and the MDT can combine the assessments of surgeons, radiologists, oncologists, and stoma therapists, among others (33). The rate of MDT participation is positively correlated with adherence to treatment, especially in complex cases and recurrences (6, 34).

MDT involvement is more frequent in CRC patients receiving chemotherapy, targeted therapy and Chinese herbal medicine for treatment or palliative care, and there were differences in the intrinsic need for multidisciplinary collaboration across treatment modalities.MDT discussions play an important role in selecting appropriate treatment regimens, integrating patients’ individualised needs and response to treatment, improving the precision and efficacy of treatment, and decreasing the incidence of adverse effects (34). Chemotherapy regimens often involve combinations of multiple drugs, requiring strict monitoring of toxicities and dynamic adjustment of the treatment regimen. Dynamic changes in carcinoembryonic antigen (CEA) in conjunction with imaging response can coordinate the number of cycles of chemotherapy with the timing of surgery, resulting in a higher success rate of translational therapy (35). Targeted therapies need to be combined with molecular pathology testing to screen for potential benefit. Collaboration between medical oncology and pathology can optimise targeted drug selection and monitor resistance mechanisms. Complex symptoms and pain are often associated with TCM or palliative care patients, and comprehensive supportive care can be provided by combining nutritional, psychological and interventional departments (36).

The impact of quality of life was analysed in relation to the impact of the MDT. There is a positive association between MDT involvement and quality of life in CRC patients, and the difference in the social dimensions in particular was close to 0.05, so that the MDT may have a positive effect in improving the social quality of life.

## 5 Conclusions

This nationwide multicenter survey revealed that access to MDT for Chinese patients with intermediate and advanced CRC is influenced by factors such as regional distribution, choice of institution, patient and treatment characteristics, and that receiving MDT care is associated with a better quality of life. In the future, policy guidance, standardized management, and the construction of medical consortia can be used to eliminate regional differences, strengthen the MDT service level of tertiary hospitals to promote the sinking of medical services, and develop differentiated education strategies for patients with different educational backgrounds.

Limitations of this study: Due to the cross-sectional design, it was not possible to assess long-term outcomes, and recall bias may have been present. In terms of statistical methods, traditional logistic regression did not account for clustering effects by hospital or region, which may have led to an underestimation of standard errors; additionally, the variable selection strategy based on univariate analysis may have overlooked potential confounding variables. Future studies should focus on improving the diagnosis of multicollinearity in the model, as well as assessing its discriminatory power and calibration, and should conduct internal validation. Lack of data related to primary hospitals, subsequent in-depth studies related to primary hospitals are needed.

## Data Availability

The raw data supporting the conclusions of this article will be made available by the authors, without undue reservation.
